# Operationalizing Clinical Competence among Occupational Physicians: Three Domains and Five Cross-Cutting Processes Identified by Reflexive Thematic Analysis

**DOI:** 10.31662/jmaj.2025-0590

**Published:** 2026-05-08

**Authors:** Tomoari Mori, Yoshiyuki Shibata, Yoto Taguchi, Hiroyuki Koga, Hiroko Kitamura, Shoko Kawanami

**Affiliations:** 1 Department of Medical Ethics, Tokai University School of Medicine, Isehara, Japan; 2 Occupational Health Training Center, University of Occupational and Environmental Health, Japan, Kitakyushu, Fukuoka, Japan

**Keywords:** occupational physicians, clinical competence, occupational health practice, preventive and advisory medicine, clinical reasoning, qualitative research, reflexive thematic analysis

## Abstract

**Introduction:**

Occupational physicians (OPs) in Japan share a common medical foundation with other clinicians, but they primarily work in advisory and preventive settings. How this medical foundation is recontextualized and operationalized as clinical competence in everyday occupational practice remains underexplored. Clarifying this process may help to better understand how biomedical reasoning is applied to workplace decision-making beyond diagnosis and treatment.

**Methods:**

We conducted a qualitative study using reflexive thematic analysis (RTA) of semi-structured interviews with 12 OPs in Japan. Braun and Clarke’s six-phase framework guided iterative coding, theme development, and interpretive synthesis. Credibility was enhanced through reflexive, iterative analytic procedures.

**Results:**

Analysis identified three interrelated practice domains: (1) statutory occupational health duties, including fitness-for-work and return-to-work judgment, (2) advice and negotiation with stakeholders, and (3) individualized support and follow-up, and five cross-cutting processes: role adaptation, reinterpretation of prior clinical expertise, responsiveness to legal and organizational contexts, collaborative negotiation, and reflective learning. Across these domains, OPs selectively mobilized clinical knowledge to address often undifferentiated health concerns, translating biomedical reasoning into context-sensitive judgments, advice, and coordination under workplace constraints. Despite substantial variability in how clinical competence was understood, recurring operational patterns were observed across participants’ accounts.

**Conclusions:**

Clinical competence in occupational medicine was not defined by fixed standards or uniformly shared expectations. Nevertheless, practice remained operationally coherent through recurring patterns across three domains and five processes, through which OPs selectively mobilized and translated clinical reasoning in response to role-related, organizational, and regulatory demands. Rather than prescribing a single competence standard, these findings provide an empirical basis for understanding how general medical training is adapted for preventive and advisory practice in occupational health, and may inform reflective, case-based learning in heterogeneous training settings.

## Introduction

Occupational physicians (OPs) in Japan share the same undergraduate and postgraduate medical training as other clinicians, forming a common medical foundation. However, their career entry points vary: some enter occupational health practice immediately after residency, whereas others transition following years of clinical experience. As a result, OPs bring heterogeneous clinical backgrounds into occupational settings, where their work is primarily non-curative, advisory, and preventive. In these roles, physicians are required to make judgments and recommendations at the interface of clinical medicine, legal obligations, and organizational management, often without direct responsibility for diagnosis or treatment ^(1), (2)^. This differentiation raises a fundamental question: what constitutes “clinical competence” when physicians operate outside conventional curative practice?

International frameworks, such as the Canadian Medical Educational Directives for Specialists and the Accreditation Council for Graduate Medical Education competencies, conceptualize physician competence as an integration of multiple roles, including medical expertise, communication, collaboration, and professionalism ^(3), (4)^. In contrast, occupational health education in Japan has traditionally emphasized regulatory compliance and biomedical knowledge, while the practical integration of broader professional competencies into everyday occupational practice remains insufficiently articulated ^(1)^. As OPs increasingly address psychosocial issues, return-to-work coordination, and complex stakeholder interactions, there is a growing recognition of the need to better understand how clinical reasoning supports advisory and preventive decision-making in workplace settings ^(5), (6), (7), (8)^.

Previous qualitative studies have examined discrete aspects of OP practice, including communication with employers ^(9)^, fitness-for-work assessments ^(10)^, and ethical conflicts arising in occupational contexts ^(11)^. However, relatively little attention has been paid to how these activities are underpinned by integrated clinical reasoning processes in everyday occupational practice, particularly in contexts where physicians are not primarily responsible for diagnosis or treatment. Much of the existing literature describes competence in terms of identifiable knowledge or skills, but offers limited insight into how such competence is enacted and adjusted in practice under conditions of uncertainty, multiple stakeholder expectations, and organizational constraints.

In this study, we use the term “clinical competence” in a situated and analytic sense that is narrower than the broader construct of physician competence described in international frameworks. Rather than encompassing the full spectrum of integrated physician roles, our focus is on how abilities that physicians themselves recognize as “clinical,” developed through prior diagnostic and therapeutic experience, are recontextualized and mobilized in advisory and preventive occupational health practice. Accordingly, this analysis does not aim to propose a normative or fixed competence standard. Instead, it provides an empirically grounded clarification of how self-perceived clinical competence is selectively mobilized, translated, and calibrated to role boundaries, organizational constraints, and legal responsibilities in everyday occupational health practice.

Perspectives from adaptive expertise ^(12)^ and workplace-based learning ^(13), (14)^ provide useful lenses for interpreting how professional capability develops through experience, reflection, and iterative negotiation with contextual demands. These perspectives emphasize that competence is not merely applied, but is continually shaped through practice. Within this theoretical context, it becomes important to examine how OPs recontextualize general medical training and how they operationalize clinical reasoning in preventive and advisory workplace settings, where direct diagnosis and treatment are not the primary objectives.

Accordingly, this study aimed to elucidate how OPs reconstruct and operationalize clinical competence in routine workplace settings. Using reflexive thematic analysis (RTA), we examined both (i) variability in how clinical competence is understood and (ii) recurring operational patterns through which clinical reasoning is translated into preventive and advisory decision-making.

## Materials and Methods

### Study design

This study adopted a qualitative design based on RTA, as developed by Braun and Clarke ^(15)^. The approach was selected to capture the interpretive and context-dependent ways in which OPs understand and enact clinical competence in everyday practice. RTA emphasizes the researcher’s reflexive engagement with the data and the construction of meaning patterns through iterative interpretation rather than through mechanical coding. No a priori theoretical framework was imposed at the start of the analysis; themes emerged through repeated reading, coding, and team discussion.

### Participants and sampling

Participants of this study included twelve OPs (eight men and four women) currently practicing in Japan. Inclusion criteria were: (1) completion of medical education and initial postgraduate training, (2) current involvement in occupational health activities, and (3) willingness to participate in an in-depth interview. Purposive sampling was used to ensure heterogeneity across employment types―full-time corporate OPs, part-time contract OPs serving multiple workplaces, and academic faculty members involved in occupational health education. Participants’ years of experience in occupational health ranged from two to twenty-five, encompassing those who entered the field directly after residency and those who transitioned from clinical specialties such as internal medicine, psychiatry, and emergency medicine. A summary of participant characteristics is presented in Table 1.

**Table 1.** Participant Characteristics (n = 12; Occupational Physicians).

Participant ID	Gender	Age group	Career path after initial residency	Clinical experience (yrs)	Full-time OP experience (yrs)	Contract OP experience (yrs)
P1	F	40s	Hospital physician → Occupational health (transition after clinical career)	> 15	3-5	> 10
P2	M	40s	University hospital → OP (center for occupational training)	> 10	3-5	> 10
P3	M	30s	Clinical practice → Corporate OP position	5-10	< 3	3-5
P4	F	50s	Acute-care medicine → Industrial medical department	> 20	> 10	> 10
P5	F	30s	Direct entry after residency	3-5	< 3	3-5
P6	F	40s	Psychiatry → Occupational health and education	> 15	3-5	> 10
P7	M	40s	Clinical medicine → University-affiliated OP and research	> 10	3-5	5-10
P8	M	30s	Emergency medicine → Corporate OP	5-10	< 3	3-5
P9	M	50s	Hospital specialist → Full-time corporate OP	> 20	> 10	> 10
P10	M	40s	Rehabilitation → Corporate OP	> 10	3-5	5-10
P11	M	50s	Internal medicine → Independent occupational health consultant	> 20	> 10	> 10
P12	M	40s	Public health and hospital practice → Regional OP network	> 15	3-5	5-10

F: female; ID: identification; M: male; OP: occupational physician. Age group and years of experience are presented in broad ranges to minimize the risk of participant re-identification. All participants completed standard undergraduate and postgraduate medical education, and held national licensure as physicians in Japan. Each participant is treated as a unique analytic case (P1-P12), corresponding to interview quotations presented in the Results section.

Potential participants were recruited using purposive sampling combined with snowball sampling through professional networks related to OP education. Individuals who expressed interest received an information sheet and were subsequently contacted by email to schedule an interview.

To consider sample sufficiency, we drew on both the concept of theoretical saturation and the notion of information power in qualitative research. During iterative analysis, we monitored whether new interviews generated substantively new practice domains or croscutting processes. As the analysis progressed, later interviews mainly elaborated or nuanced previously identified patterns, and no fundamentally new thematic structures emerged. Given the focused research aim and the heterogeneity of participants’ backgrounds and career trajectories, we judged that the dataset provided sufficient information power and conceptual depth to support this analysis.

### Data collection

Semi-structured interviews were conducted between January and October 2023 by the first author (TM), an OP trained in qualitative research and medical ethics. Interviews were conducted either face-to-face or via secure video conferencing, each lasting approximately 60-90 minutes. The interview guide (Supplementary File 1) included open-ended questions addressing (1) concrete workplace situations involving clinical or ethical judgment, (2) interactions and negotiations with workers, employers, and other professionals, and (3) application or adaptation of clinical knowledge within preventive and organizational contexts. All interviews were conducted in Japanese, audio-recorded with consent, and transcribed verbatim. Field notes and analytic memos were written immediately after each session to capture contextual details and reflexive insights. Although the interview guide focused on eliciting concrete workplace episodes rather than predefined domains, the three practice domains were analytically derived during the analysis phase by grouping recurrent patterns in how participants described their clinical involvement in occupational health practice.

### Data analysis

Data were analyzed using RTA following the six-phase framework of Braun and Clarke ^(15)^: (1) familiarizing through repeated reading, (2) generating initial codes inductively from meaningful segments, (3) constructing candidate themes, (4) reviewing and refining themes through iterative team discussion, (5) defining and naming themes, and (6) producing the analytic narrative linking empirical insights to occupational health practice and professional development. The first author (TM) conducted the primary coding using NVivo (Release 12 Plus, QSR International) ^(16)^, and themes were refined through reflexive dialogue among the research team. Credibility was enhanced through continuous reflexive memoing, peer debriefing, and maintenance of an analytic audit trail (Supplementary File 2). Peer debriefing involved iterative discussions within the multidisciplinary research team, in which preliminary codes and candidate themes were critically examined and refined. Rather than seeking inter-rater reliability, these discussions aimed to enhance interpretive transparency and coherence, in line with the principles of RTA. The five cross-cutting processes were identified as recurring mechanisms that appeared across all three practice domains and multiple participants, rather than as domain-specific activities. Member-checking was performed with three participants to confirm the contextual resonance and plausibility of the interpretations, rather than to seek literal agreement with specific thematic labels ^(17)^. Thick description was used to preserve contextual detail when presenting quotations ^(18)^.

### Researcher reflexivity

The first author (TM) is a physician with clinical experience in emergency and occupational medicine, which supported rapport building and interpretive sensitivity to workplace constraints. Co-authors (YS, YT, HK, HKi, and SK) brought complementary backgrounds in occupational health, clinical education, and organizational psychology. The multidisciplinary team engaged in iterative discussions to challenge assumptions and to enhance analytic depth, maintaining both insider and outsider perspectives throughout the process.

### Artificial intelligence (AI)-assisted language editing

OpenAI ChatGPT (GPT-5.1) was used solely to improve the clarity and readability of English expressions in the manuscript. It was not used for study design, data analysis, or interpretation of the findings. All AI-assisted edits were reviewed, verified, and approved by the authors, who retain full responsibility for the content.

### Ethical considerations

The study was approved by the Institutional Review Board of the University of Occupational and Environmental Health, Japan (Approval No. R4-072), and was conducted in accordance with the Declaration of Helsinki. All participants received written and verbal explanations of the study objectives, confidentiality measures, and their right to withdraw, and they provided written informed consent, including permission to publish anonymized quotations. Identifying details were removed or generalized during transcription and reporting to ensure participant confidentiality.

## Results

### Variability in understandings of “clinical competence”

Participants’ accounts showed substantial variability in how “clinical competence” was understood in occupational health practice. For some, clinical competence was viewed as necessary for credible judgment and communication, particularly when anticipating health trajectories rather than when assessing a static condition: “In the workplace, what matters is how a condition may worsen… That sense changes how you talk to the worker and the workplace” (P1). Relatedly, several participants framed their “clinical” contribution not as making a definitive diagnosis, but as generating differential possibilities and guiding appropriate referrals: “It’s not that we make a definitive diagnosis… we can suggest which specialty the person should see… [and] widen the possibilities” (P2).

In contrast, other participants explicitly questioned whether advanced clinical competence is necessary in occupational practice, and instead emphasized an initial assessment-and-connection role: “I don’t think ‘being good at clinical medicine’ strongly correlates with ‘being good as an occupational physician’… what matters is first-touch assessment… and connecting [workers] to the appropriate resources” (P11). This position was also linked to concerns about role boundaries. Some participants noted that OPs are not expected to provide full diagnostic or treatment decisions, and that excessive clinical involvement could blur professional roles and complicate collaboration with attending physicians: “As occupational physicians, we are not diagnosing or treating in the same way as clinicians; our role is different, and that distinction matters” (P5).

Taken together, these accounts indicate that participants did not converge on a single definition of clinical competence in occupational health, including its necessity, scope, or depth. Rather than reflecting inconsistency, this diversity highlighted differences in how clinical knowledge was positioned and mobilized in practice. Notably, across these varying perspectives, participants commonly described relying on selective use of medical knowledge: identifying what was relevant in a given situation, accessing information when needed, and calibrating their clinical engagement to role boundaries and workplace constraints.

### Recurring operational patterns: three domains and five cross-cutting processes

Despite the variability described above, the analysis identified recurring patterns in how participants described using medical knowledge and clinical reasoning in occupational health practice. These patterns were organized into three interrelated practice domains and five cross-cutting processes that recurred across participants’ accounts, regardless of how “clinical competence” itself was defined. Participant identifiers (P1-P12) are used for quotations.

### Three practice domains

Participants described enacting clinical reasoning across three interrelated domains (Table 2).

**Table 2.** Summary of Three Practice Domains and Five Cross-Cutting Processes.

Domain/process	Description (summary)
Domain 1: Statutory occupational health duties	Translating clinical reasoning into statutory occupational health responsibilities, such as work-capacity judgment and related preventive measures, by integrating health status with job demands, workplace feasibility, and anticipated trajectories.
Domain 2: Stakeholder negotiation and advice	Translating medical considerations into actionable advice through negotiation with workers, supervisors, and human-resources staff, balancing organizational feasibility, worker autonomy, and professional accountability.
Domain 3: Individualized support and follow-up	Coordinating referral, follow-up, and preventive support for workers facing psychosocial or chronic conditions, while maintaining appropriate care boundaries and feasibility in workplace settings.
Process 1: Role adaptation	Reframing the physician’s role from curative, episode-based practice to preventive and context-dependent occupational reasoning.
Process 2: Reinterpretation of prior clinical expertise	Selectively mobilizing prior diagnostic and therapeutic experience into functional assessment, risk anticipation, and advisory communication appropriate to occupational contexts.
Process 3: Legal and organizational responsiveness	Integrating statutory requirements and organizational rules into decision rationales and documentation while preserving medical integrity and professional boundaries.
Process 4: Collaborative negotiation	Engaging stakeholders through transparent and proportional reasoning to make recommendations workable in practice and to foster shared accountability.
Process 5: Reflective learning	Refining judgment and boundary management through reflection on prior cases, uncertainty, and emotional demands, and supporting adaptive and sustainable practice.

Domains represent arenas of occupational practice, whereas processes denote cross-cutting mechanisms through which clinical reasoning is selectively mobilized and translated into decisions.

### Domain 1: Statutory occupational health duties

Accounts described statutory responsibilities in occupational health practice in which clinical reasoning was mobilized, including fitness-for-work and return-to-work judgment, follow-up of health examination results, and mental health-related measures. Participants described integrating health status with job demands, workplace feasibility, and anticipated trajectories, emphasizing functional evaluation and risk anticipation in ongoing follow-up rather than in one-time biomedical determinations.

### Domain 2: Stakeholder negotiation and advice

Participants described advising and negotiating with supervisors, human-resources staff, and external clinicians. They reported translating medical considerations into recommendations that could be communicated and implemented within workplace constraints, including responding to requests for simplified decisions (e.g., fit/unfit) by articulating conditional reasoning and by clarifying role boundaries.

### Domain 3: Individualized support and follow-up

Participants described individualized support that included coordination and follow-up (e.g., arranging referrals, monitoring progress after referral, and clarifying warning signs), as well as activities aimed at preventive preparedness within workplaces. These accounts emphasized supporting safety and feasibility without replacing curative care.

### Five cross-cutting processes

**Figure 1.**
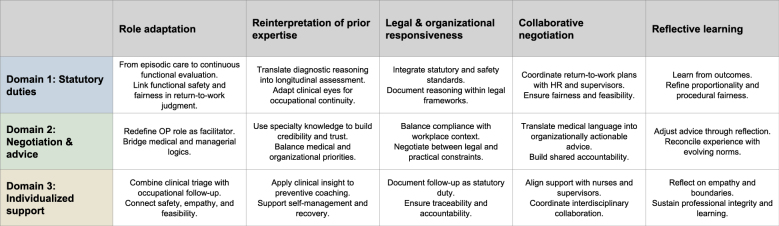
Operational structure of clinical competence in occupational physicians (three domains × five processes). This figure summarizes the recurring operational structure through which occupational physicians selectively mobilize and translate clinical reasoning into everyday practice. The structure comprises three interrelated practice domains―(1) statutory occupational health duties, (2) advisory and negotiatory interactions with stakeholders, and (3) individualized support and follow-up―and five cross-cutting processes identified across participants’ accounts. HR: human resources; OP: occupational physician.

Across the three domains, five processes recurred in participants’ descriptions of how clinical reasoning was made workable in occupational settings (Table 2, Figure 1).

### Role adaptation

Participants described shifting from curative, episode-based clinical work toward preventive, context-dependent occupational reasoning, including reframing what constituted an appropriate “medical” contribution in workplace settings.

### Reinterpretation of prior clinical expertise

Participants described translating prior specialty experience into occupationally relevant reasoning, such as functional assessment, risk anticipation, and advisory dialogue.

### Legal and organizational responsiveness

Participants described incorporating statutory requirements and workplace rules into their rationales and documentation, while maintaining professional boundaries and medical integrity.

### Collaborative negotiation

Participants described engaging stakeholders through explanation, alignment, and communication to make recommendations workable in practice and to coordinate responsibilities.

### Reflective learning

Participants described revisiting prior cases and uncertainties to refine decision thresholds, boundary management, and proportional engagement over time.

### Cross-cutting nature of the five processes

These processes were described as cross-cutting rather than as domain-specific. Participants often referred to the same process (e.g., legal responsiveness or collaborative negotiation) in different domains (e.g., return-to-work judgment and stakeholder advice), indicating that the processes functioned as recurring mechanisms through which medical reasoning was translated into occupational decisions (Figure 1).

Within this operational structure, participants described selectively mobilizing clinical knowledge in recurring ways. Across domains, they reported (i) anticipating plausible courses and warning signs, (ii) generating differential possibilities sufficient to guide referral, (iii) translating medical considerations into conditional recommendations that organizations could implement, and (iv) communicating uncertainty explicitly while preserving credibility and professional boundaries. These ways of using clinical knowledge were not confined to a single domain or process, but they appeared across the three domains through the five cross-cutting processes, illustrating how clinical reasoning content was filtered and reconfigured to support preventive and advisory decision-making in occupational settings.

## Discussion

This study examined how OPs in Japan operationalize “clinical competence” in everyday practice that is primarily advisory and preventive rather than treatment-oriented. Two findings are central. First, participants differed substantially in what they considered to “count” as clinical competence, including its necessity, scope, and depth, as illustrated in the opening segment of the Results section. Second, despite this conceptual variability, participants described recurring ways of organizing and applying clinical reasoning across three practice domains and five cross-cutting processes, as detailed in the subsequent segment of the Results section.

Importantly, the aim of this study was not to propose a normative or fixed competence standard. Rather, the analysis offers an empirically grounded clarification of how competencies regarded as “clinical” are selectively mobilized, translated, and calibrated to organizational constraints, legal responsibilities, and professional role boundaries in everyday occupational health practice. The findings should therefore be understood as an operational account derived from participants’ sense-making and practice narratives, rather than as a prescriptive formulation of competence.

Taken together, these findings suggest that occupational practice can remain operationally coherent even in the absence of a single, shared competence threshold. Rather than being sustained by uniform standards, coherence appeared to be maintained through reproducible ways of translating biomedical reasoning into workplace judgments, advice, and coordination under organizational and regulatory constraints.

### Variability is not simply a deficit: why conceptual boundaries diverge

Participants’ accounts rarely converged on a stable threshold of clinical knowledge or experience that would define competence for all OPs. Some emphasized the importance of clinical experience for anticipating trajectories, interpreting medical information, and communicating risk in a way that workplaces can act on. Others, including physicians with substantial prior clinical experience, questioned whether advanced clinical competence is necessary for occupational practice, and instead emphasized on boundary management, first-touch assessment, and appropriate referral. This divergence is understandable in Japan’s heterogeneous occupational health ecology, where the intensity and type of cases, organizational expectations, and availability of external resources differ widely across industries and regions.

Importantly, these differences should not be interpreted as inconsistency that must be eliminated. OPs work at the intersection of clinical medicine, legal duties, and organizational management, and the appropriate level of clinical involvement is often situation-dependent. In this interface, competence is shaped by the specific decision context, workplace feasibility, and the need to maintain professional boundaries while collaborating with treating clinicians. From this perspective, the absence of a single competence formulation is not a conceptual weakness, but rather an empirical feature of practice that this study aims to describe.

### How practice stays coherent: translation of clinical reasoning in workplace settings

Though understandings of clinical competence varied, participants’ accounts converged on how clinical reasoning was translated into practice. Across settings, OPs did not describe applying comprehensive diagnostic expertise or making definitive treatment decisions. Instead, they reported selectively mobilizing clinical reasoning in four recurring ways: (i) anticipating plausible courses and warning signs, (ii) generating differential possibilities sufficient to guide referral, (iii) translating medical considerations into conditional recommendations that organizations could implement, and (iv) communicating uncertainty explicitly while preserving credibility and maintaining professional boundaries.

These forms of reasoning were distributed across the three practice domains and were enacted through the five cross-cutting processes identified in the Results section. In this sense, translation did not refer merely to simplification, but to the reconfiguration of biomedical reasoning so that it became proportionate, actionable, and accountable within workplace constraints. This perspective helps explain how occupational practice can remain coherent, even when OPs differ in how much clinical expertise they consider necessary.

### Clinical competence as selective mobilization of clinical reasoning

Across accounts, what mattered was not maximal clinical involvement but it was the selective mobilization of clinical reasoning. At an analytic level, clinical competence can thus be understood as the context-sensitive capacity to selectively mobilize and translate clinical reasoning into advisory and preventive decision-making within organizational and regulatory constraints. Participants emphasized identifying which aspects of medical knowledge were relevant to the decision at hand, accessing information when needed, and calibrating the depth of reasoning to role boundaries and context.

### Implications for professional development in Japan

Although this study does not propose assessment standards, it offers practice-oriented implications for the education and continuing professional development of OPs, including those involved in Japan Medical Association training programs. The three-domain, five-process structure can function as a shared analytic language for case-based discussion and for supervision focused on real-world constraints: which clinical considerations were treated as relevant, how uncertainty was communicated, how role boundaries and referral decisions were managed, and how recommendations were made workable for stakeholders.

In practice, this structure may be used as a reflective scaffold in training settings, enabling learners with heterogeneous clinical backgrounds to systematically revisit their reasoning across cases and to translate experiential knowledge into context-sensitive advisory and preventive actions, without reducing competence to checklist-based behaviors.

### Future research

Future research could strengthen the empirical basis of this operational account through longitudinal studies on the development of selective mobilization, comparative analyses across organizational contexts, and mixed-methods investigations linking these operational patterns to outcomes such as perceived legitimacy of decisions or feasibility of workplace accommodations.

### Limitations

This study has several limitations. Findings are based on interviews with a small sample of OPs practicing in Japan, and may not transfer directly to settings with different legal frameworks, training pathways, or service models. The data reflect self-reported experiences and cannot fully capture real-time decision processes or stakeholder perspectives. Observational and multi-perspective studies would complement these findings. As with all RTA, the analytic framework presented here is interpretive and provisional, and should be treated as a descriptive account open to refinement.

### Conclusions

This study clarifies how OPs in Japan operationalize “clinical competence” in everyday practice that is primarily advisory and preventive rather than treatment-oriented. The findings indicate that, although OPs differ substantially in how they define the necessity, scope, and depth of clinical competence, occupational practice remains operationally coherent through shared ways of selectively mobilizing and translating clinical reasoning under organizational and regulatory constraints. By empirically describing three practice domains and five cross-cutting processes, this study provides a descriptive account of how general medical training is recontextualized for occupational health practice without presupposing a single competence standard. In this study, therefore, clinical competence is analytically conceptualized, in an operational sense, as the practice capability to selectively mobilize and translate clinical reasoning into advisory and preventive workplace decision-making, rather than as a fixed or normative definition. This perspective may serve as a useful foundation for future empirical, educational, and professional discussions on clinical competence in occupational medicine.

## Article Information

### Author Contributions

Conceptualization and study design: Tomoari Mori. Data collection and analysis: Tomoari Mori (lead); Yoshiyuki Shibata, Yoto Taguchi, Hiroyuki Koga, Hiroko Kitamura, Shoko Kawanami (contributors). Methodology and validation: Tomoari Mori, Yoshiyuki Shibata, Yoto Taguchi, Hiroyuki Koga, Hiroko Kitamura, Shoko Kawanami (iterative discussions and reflexive review). Writing―original draft: Tomoari Mori. Writing―review & editing: Yoshiyuki Shibata, Yoto Taguchi, Hiroyuki Koga, Hiroko Kitamura, Shoko Kawanami. Supervision: Tomoari Mori. All authors approved the final manuscript.

### Conflicts of Interest

None

### Ethical Approval

Approved by the Institutional Review Board of the University of Occupational and Environmental Health, Japan (Approval No. R4-072).

## Supplement

Supplementary Material
