# Supplementary material for: Operationalizing Clinical Competence among Occupational Physicians: Three Domains and Five Cross-Cutting Processes Identified by Reflexive Thematic Analysis
Source: JMA J. 2026 May 8;9(4):778–86. doi: 10.31662/jmaj.2025-0590 (PMC13453497; doi:10.31662/jmaj.2025-0590)
Supplement: Supplementary file 1 [file 2433-3298-9-4-0778-s001.pdf]

Supplementary File 1. Interview Guide (Japanese – English)

Note: This guide is provided as interviewer script. Participants see only the study information and consent materials.

日本語（Japanese）	English
**導入**	**Introduction**
【確認事項】<br>本日のインタビューは録音させていただきます。記録は匿名化され、研究目的以外には使用されません。途中で中断や質問をやめることも自由にできます。ご了承いただけますか？	[Confirmation]<br>This interview will be audio-recorded. The recordings will be anonymized and used only for research purposes. You are free to pause or stop answering at any time. Do I have your consent?
【質問】<br>産業医として勤務されている中で、印象に残っている臨床能力を用いた業務や対応について教えてください。	[Question]<br>In your work as an occupational physician, could you tell me about a task or response where your clinical competence was particularly memorable?
【追加質問（プロービング）】<br>それはいつごろ、どのような状況でしたか？／そのとき、どのような対応をされましたか？／その経験からどのようなことを感じましたか？	[Probing prompts]<br>When was it, and in what kind of situation? / How did you respond at that time? / What did you take away from that experience?
**臨床能力の理解**	**Understanding of Clinical Competence**
【質問】<br>「臨床能力」という言葉を聞いて、どのようなイメージがありますか？	[Question]<br>When you hear the term “clinical competence,” what comes to mind?

【追加質問（プロービング）】<br>実際の業務でそれを感じる場面はありますか？／診療を行わない場面でも、臨床的判断が求められると感じたことはありますか？	[Probing prompts]<br>Are there moments in your daily work where you sense this competence? / Have there been situations outside direct medical care where clinical judgment was still required?
**臨床能力の活用場面**	**Contexts of Applying Clinical Competence**
【質問】<br>日常業務の中で、ご自身が医師としての専門性や判断力を発揮していると感じる場面について教えてください。	[Question]<br>In your daily practice, can you describe situations where you feel you exercise your expertise and judgment as a physician?
【追加質問（プロービング）】<br>それはどのような業務でしたか？／どのような判断や配慮を行いましたか？／その際、どのような知識や経験が役立ちましたか？	[Probing prompts]<br>What kind of task was it? / What kinds of judgments or considerations did you make? / What knowledge or experiences were particularly helpful?
**臨床能力の認識と影響要因**	**Perceptions and Influencing Factors**
【質問】<br>臨床能力の認識や活用に影響を与えた経験や状況について教えてください。	[Question]<br>Could you tell me about experiences or circumstances that have influenced how you recognize and apply clinical competence?
【追加質問（プロービング）】<br>外部の医療機関や臨床医と連携された経験について教えてください。／どのような状況で連携が必要になりましたか？／具体的にどのようなやり取りをされましたか？／その際、ご自身の臨床的判断や専門性がどのように活かされたと感じましたか？／連携の中で、どのような能力	[Probing prompts]<br>Have you had experiences collaborating with external medical institutions or clinicians? / In what situations was such collaboration necessary? / What specific interactions took place? / How did you feel your own clinical judgment and expertise were utilized? / What abilities did you feel were needed during such collaboration? / Did this experience change how you perceive or use clinical competence?

が必要だと感じましたか？／その経験を通して、臨床能力の認識や使い方に変化があったと感じますか？／ご自身の臨床経験や専門性は、この連携にどう影響しましたか？	/ How did your clinical background or expertise influence this collaboration?
**終了前の確認**	**Final Check**
【質問】<br>本日のお話の中で、特に強調しておきたいことや、他にお伝えになりたいことがあれば教えてください。	[Question]<br>Is there anything from today’s discussion that you would particularly like to emphasize, or anything else you would like to add?
【追加質問（プロービング）】<br>本日のインタビューを通して、改めて感じたことはありますか？	[Probing prompt]<br>Did you come to any new realizations through this interview?
【メンバーチェッキング予告】<br>本日の内容を要約し、後日確認のためにご連絡することがあります。	[Member-checking notice]<br>We may contact you later with a summary of today’s discussion for confirmation.

Supplementary File 2. Analytic transparency

(Reflexive Thematic Analysis, summary)

Data preparation & management

All interviews were audio-recorded and transcribed verbatim. Transcripts were anonymized before analysis. Coding was conducted manually; transcripts and memos were stored securely.

Analytic steps (RTA)

We followed reflexive thematic analysis, moving iteratively across familiarization, code generation, theme development, theme review/refinement, and naming/definition. Decisions were logged in analytic memos.

Reflexivity: The team included occupational physicians and qualitative researchers. We documented potential preconceptions (e.g., feasibility-oriented bias) and reflected on their influence through memoing.

Credibility strategies

Credibility was enhanced through peer debriefing at key milestones, maintenance of an analysis log, and targeted member checking focused on factual accuracy.

Information power: Adequate given the focused aim, sample heterogeneity (clinical background, employment status, and sector), and interview richness (eight one-to-one; two dyadic sessions).

Theme development chain (excerpt)

Theme	Subtheme / process marker	Illustrative quote (P#)	Analytic note
Making plans workable	Organizational alignment	“We had to adapt to the workplace rhythm…” (P7)	Linked to capacity rhythms; aligns with statutory legibility
Operationalizing legal requirements	Legibility to statutes	“My plan had to be justifiable under the law.” (P2)	Distinguishes legal vs operational constraints

Audit trail (index): Analysis log entries (dates); decision memos (theme merge/split); member-checking summaries. Full materials available upon request.

Identification of cross-cutting processes

During theme development, candidate themes and process labels were iteratively reviewed to determine whether they functioned as domain-specific activities or as cross-cutting processes. A process was considered “cross-cutting” when it met the following criteria: (1) it recurred across all three practice domains, rather than being confined to a single domain; (2) it was described by multiple participants with differing clinical backgrounds and employment settings; and (3) it operated as a mechanism for translating clinical reasoning into occupational action, rather than as a task-specific outcome.

Several initial candidates were discussed during analysis. Some were ultimately integrated into broader processes (e.g., emotionally demanding aspects of practice were incorporated into reflective learning), while others were not retained because they did not consistently appear across domains or functioned primarily as contextual conditions rather than operative processes. Through this iterative refinement, five cross-cutting processes were identified as analytically distinct yet recurrent across participants’ accounts.

Illustrative quotations for cross-cutting processes

The following excerpts illustrate how each cross-cutting process was articulated in participants’ accounts. These quotations are not intended to exhaustively represent all instances but to provide representative examples demonstrating how the processes were grounded in the data.

Process 1: Role adaptation

“In occupational health, it’s not about treating one episode. You have to keep thinking about whether the person can continue working safely over time.” (P11)

Process 2: Reinterpretation of prior clinical expertise

“Clinical experience doesn’t mean applying hospital medicine as it is. It’s about knowing how far you can go, and where you should stop.” (P10)

Process 3: Legal and organizational responsiveness

“If what I suggest doesn’t fit the legal framework, it simply won’t work in the workplace.” (P5)

Process 4: Collaborative negotiation

“Just saying ‘this is medically risky’ doesn’t move anything. I have to explain it in a way that supervisors and HR can actually act on.” (P1)

Process 5: Reflective learning

“Looking back, I realized that responding to every request wasn’t necessary. That reflection changed how I handle similar cases now.” (P7)
